# Disseminated life threatening *Nocardia otitidiscaviarum* infection in a young female with newly diagnosed systemic lupus erythematosus, case report and review of literature

**DOI:** 10.1016/j.idcr.2021.e01265

**Published:** 2021-09-01

**Authors:** Jabeed Parengal, Seham Mohsin Alebbi, Manal Mahmoud Mohamed Hamed, Hosam Mohammed Alqatami, Fatma Ben Abid

**Affiliations:** a Department of Medicine, Division of Infectious Diseases, Hamad Medical Corporation, Doha, Qatar; b Department of Medicine, Division of Rheumatology, Hamad Medical Corporation, Doha, Qatar; c Department of Microbiology, Hamad Medical Corporation, Doha, Qatar; d Neurosciences Institute, Hamad Medical Corporation, Doha, Qatar; e Weill Cornell Medical College, Doha, Qatar

**Keywords:** *Nocardia otitidiscaviarum*, Intracranial aneurysm, Systemic lupus erythematosus, Brain abscess, Pneumonia, Pustule

## Abstract

Infection due to *Nocardia* is reported mainly in immunocompromised patients. It usually presents as a pulmonary or disseminated disease with a predilection for the brain. Infections are a rare etiology of intracranial vascular aneurysms. Herein we report a case of disseminated *Nocardia otitidiscaviarum* (*N*. *otitidiscaviarum*) in a young female newly diagnosed with systemic lupus erythematosus (SLE) complicated by the development of an infectious intracranial aneurysm. To the best of our knowledge this is the fourth case of nocardial infection-related intracranial aneurysm and the second case of *N. otitidiscaviarum* infection to be reported in a patient with systemic lupus erythematosus. Features of previously reported *N. otitidiscaviarum* related intracranial aneurysm are reviewed.

## Introduction

Nocardiosis is considered an opportunistic infection affecting immunocompromised patients. The species *N. otitidiscaviarum* was considered a soil saprophyte until the first human infection was described 50 years after the identification of the species. Diabetes mellitus, human immunodeficiency virus (HIV) infection, and post-transplant status were the most common risk factors identified in previously described N. *otitidiscaviarum* infections. Only one reported case of infection due to this unusual species was associated with systemic lupus erythematosus (SLE).

To date, there are only 3 reported cases of intracranial artery aneurysm caused by nocardial infection. We are reporting a case of *N. otitidiscaviarum* infection in a young female with SLE presenting with disseminated nocardiosis. She had multiple complications including rupture of a cerebral artery aneurysm and pneumothorax.

## Case presentation

**Fig. 1:**
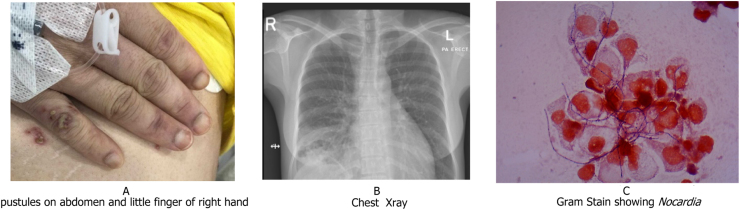
(A) pustules on abdomen and little finger of right hand (B) Chest Xray, (C) Gram Stain showing *Nocardia*.

A 29-year-old female was newly diagnosed with systemic lupus erythematosus (SLE). She had multi-organ involvement including autoimmune hemolytic anemia, cerebritis, lupus nephritis, cardiomyopathy with an ejection fraction of 33% and non-specific interstitial pneumonia. The patient was started on mycophenolate mofetil 1000 mg twice daily, hydroxychloroquine 200 mg once daily, and prednisolone 40 mg daily which was planned to be tapered over 8 weeks. She had to stay in hospital for 2 weeks due to the involvement of multiple organs and the slow response to treatment. Two weeks after her discharge from the hospital she started to experience a dry cough followed by fever, progressive shortness of breath, generalized body aches, nausea, vomiting, and skin rash. On examination, she looked thin and malnourished, with severe alopecia. Examination showed a temperature of 39.4 °C, heart rate 147 beats/minutes, respiratory rate of 24 cycles/minutes and blood pressure137/88 mm Hg. There were multiple skin pustules of different ages scattered over the upper limbs, lower limbs, back and abdomen (Fig. 1-A). Chest auscultation revealed reduced air entry at the right lung base and abdominal examination showed right upper quadrant tenderness. Investigations revealed normocytic normochromic anemia (hemoglobin level 6.8 g/L,normal range 12.0–15.0 g/L), leukocytosis (17.1 × 10^9^/L, normal range 4.0–10.0 10^9^/L) with neutrophilia (16.2 × 10/uL, normal range 2.0–7.0 10/uL), elevated C-reactive protein levels (218.7 mg/L, normal range 0.0–5.0 mg/L) and high procalcitonin (7.17 ng/mL). Anemia work up showed haptoglobin 518 mg/dL (normal range 30–200 mg/dL), lactic acid dehydrogenase 372 U/L (normal range 135–214 U/L), reticulocytes 1.5%. Regarding lupus disease activity, double stranded DNA antibodies was positive with titer of 25.00 IU/mL while complement 3 was 0.68 gm/L (normal range 0.90–1.80 gm/L) and complement 4 was 0.08 gm/L (normal range 0.10–0.40 gm/L). Chest X-ray showed consolidation with cavitation and pleural effusion in the right lower zone (Fig. 1-B). The fluid from skin pustules as well as sputum and blood samples were sent for microbiological studies. All cultures from sputum, broncho-alveolar lavage (BAL), skin pustules pus showed on gram stain branching, fine, delicate filaments with fragmentation, beaded gram-positive bacilli (Fig. 1-C). The modified Ziehl–Neelsen staining revealed partially acid-fast bacilli. *N. otitidiscaviarum* was identified by Matrix-Assisted Laser Desorption/Ionization-Time of Flight (MALDI-TOF). Epsilometer test (E test) was done to detect Minimum Inhibitory Concentration (MIC) of different antibiotics. The results were interpreted using the Clinical and Laboratory Standards Institute (CLSI) document M24. *N. otitidiscaviarum* isolate was susceptible to trimethoprim-sulfamethoxazole (TMP-SMX) (MIC 1 μg/mL), amikacin (MIC 2 μg/mL), ciprofloxacin (MIC 0.5 μg/mL), moxifloxacin (MIC 0.25 μg/mL) and linezolid (MIC 0.094 μg/mL). It was resistant to amoxicillin-clavulanate (MIC 32 μg/mL), ceftriaxone (MIC32 μg/mL) and clarithromycin (MIC 12 μg/mL). The MIC for meropenem was 0.38 μg/mL, but CLSI did not provide interpretation for meropenem in *N. otitidiscaviarum.*

**Fig. 2:**
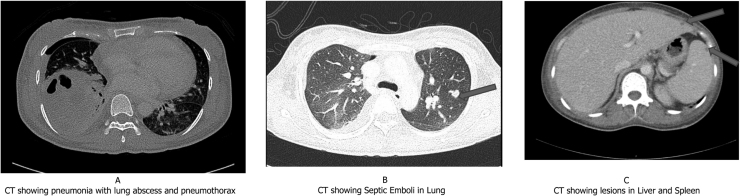
(A) CT showing pneumonia with lung abscess and pneumothorax (B) CT showing Septic Emboli in Lung (C) CT showing lesions in Liver and Spleen.

**Fig. 3:**
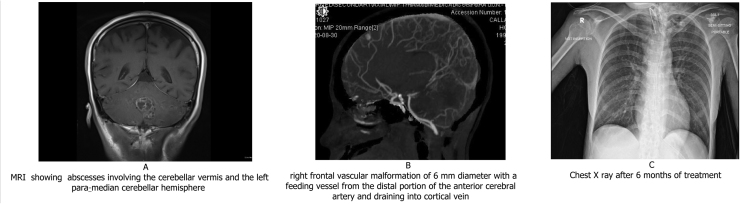
(A) MRI showing abscesses involving the cerebellar vermis and the left para-median cerebellar hemisphere (B) right frontal vascular malformation of 6 mm diameter with a feeding vessel from the distal portion of the anterior cerebral artery and draining into cortical vein (C) Chest X ray after 6 months of treatment.

A computed tomography (CT) scan of the chest showed necrotizing pneumonia with lung abscess and pneumothorax in the left lung (Fig. 2-A). Few septic emboli also were visualized in left lung (Fig. 2-B). The images also revealed multiple collections in the liver spleen and kidneys (Fig. 2-C). Magnetic resonance imaging (MRI) of the brain showed numerous supra and infratentorial brain micro-abscesses with the largest involving the cerebellar vermis and the left para-median cerebellar hemisphere. These abscesses showed central diffusion restriction, peripheral enhancement, and internal enhancing septations (Fig. 3-A). A diagnosis of disseminated nocardiosis was taken and the patient was started on intravenous meropenem meningeal dose, TMP-SMX and amikacin. Imipenem was not available in the hospital pharmacy. The MIC for meropenem was low and previous Nocardial isolates from our hospital had shown good clinical response to regimens having meropenem. Hence decision was taken to include meropenem in treatment regimen after discussion with microbiologist. Three days later, the patient’s condition rapidly deteriorated with a decrease in her level of consciousness with a Glasgow coma scale (GCS) of 6/15, there was no neurologic deficit. Elective intubation was performed, and she was put on mechanical ventilation. A repeat imaging revealed intracranial hemorrhage and right frontal vascular malformation of 6 mm of diameter with a feeding vessel from the distal portion of the anterior cerebral artery and draining into a cortical vein (Fig. 3-B). The neurosurgery team was involved and they decided to do a lifesaving decompressive craniectomy and insertion of an external ventricular drain (EVD). The patient received intravenous antibiotics for a total of eight weeks and was then shifted to oral moxifloxacin and TMP-SMX which is planned for 10 months to complete 1 year of treatment. A CT of the brain done after 6 months of treatment showed complete resolution of the brain abscesses and a repeat chest Xray (Fig. 3-C) showed clearance of the initial changes. Despite the initiation of appropriate antibiotics and all supportive measurements, the patient continued to have low GCS level and remained ventilator-dependent, tracheostomized, and fed through a nasogastric tube.

## Discussion

*Nocardia* is a genus of aerobic bacteria in the order Actinomycetales. More than 100 species of *Nocardia* are identified [1]. The name Nocardia is derived from Edmond Nocard (1850–1903), a French veterinarian who first isolated members of this taxon [2]. *Nocardia* species (N. Spp) are weakly acid-fast bacilli. They are classified as gram-positive, but many strains have alternating positive and negative areas giving them a faint beaded appearance. They exhibit a characteristic filamentous branching with fragmentation into bacillary or coccoid forms. The species *N*. *otitidiscaviarum* was first identified in 1924 and was considered a soil saprophyte [3]. The first human infection due to *N. otitidiscaviarum* was reported in 1974 and was previously named *Nocardia caviae* [4], [5]. *N. otitidiscaviarum* is an infrequent cause of human Nocardial infections. In some series of human nocardial infections, it accounted for only 0.3–2.9% of all Nocardia infections [5]. This rarity has been attributed to its low virulence and low prevalence in the soil as compared to other *Nocardia* [6]. N. spp is ubiquitous in soil, decaying vegetable matter, and aquatic environments. A study from Iran reported isolation of *Nocardia* in the water and soil samples of 19 out of 30 studied hospitals [7]. They can become airborne, and inhalation is the most common route of infection. Ingestion and cutaneous spread have also been reported as routes of entry. N. spp causes various diseases in humans as well as animals. More than 54 species have been reported to be clinically significant [8].

Many risk factors predisposing to nocardial infections have been reported in the literature. Immunosuppression due to glucocorticoid therapy, malignancy, solid organ transplant and advanced HIV infection are the leading causes of nocardiosis [10], [11], [12], [13]. In addition, diabetes mellitus, alcoholism, chronic granulomatous disease, structural lung disease, tumor necrosis factor-alpha inhibitor therapy, inflammatory bowel disease, chronic obstructive pulmonary disease and tuberculosis have been reported as risk factors for nocardial infection [14]. However, an increasing number of cases of nocardiosis have been reported in apparently immunocompetent individuals [9]. It is possible that they may have an unidentified underlying immunodeficiency.

*Nocardia* species can infect different organs. Nocardial infection usually presents as pneumonia, lymphocutaneous infection, mycetoma, brain abscess, or disseminated infection [15]. There are reports of septic arthritis [56] and bacteremia [16], [21] caused by *Nocardia*. In a large series, isolated pulmonary involvement was the most frequent presentation accounting for 39% of all cases while 34% had disseminated disease involving two or more organs and the central nervous system (CNS) was involved in 44% of those with disseminated disease. Isolated CNS and cutaneous infection accounted for 9% and 8% of cases respectively. [15] The most common manifestation of CNS infection is brain abscess. There have been three reported cases of *Nocardia causing* aneurysms in intracerebral arteries [17], [18], [19]. One of these three patients had a background of SLE and was getting steroids while another one had multiple myeloma. All three of them responded well to surgical management along with a combination of antibiotics. The characteristics of these three cases are summarized in (Table 1). To the best of our knowledge, there are 54 reported cases of human infections due to *N. otitidiscaviarum*. The details were reviewed and summarized in (Table 2).

**Table 1:** Characteristics of the three reported cases of Nocardia associated intracranial aneurysm.

Case number	Age	Sex	Comorbidity	Immunosuppressive drugs	Presentation	Coexisting pathology	Diagnostic method	Site of aneurysm	Subsequent procedure	Antibiotic regimen
1	60	Male	None	None	headaches, fatigue, memory loss, and behavioral abnormalities for 2–3 weeks before admission	Abscess	stereotactic aspiration of the abscess	internal carotid aneurysm	underwent drainage of the abscess with subsequent resection of the infected aneurysm.	6 weeks of ceftriaxone and high-dose trimethoprim–sulfamethoxazole (TMP-SMX)
2	69	Male	Multiple myeloma	Bortezomib	Thoracic empyema alteration of consciousness with grade 3/5 right upper and lower extremity weakness	subarachnoid hemorrhage and multiple scattered small rim-enhancing lesions	Emergency clipping of the aneurysm which revealed necrotic aneurysm and thrombus occlusion at the left middle cerebral artery	Left middle Cerebral artery	.	(TMP-SMX,15 mg/kg/day) and ceftriaxone (4 g/day)
				Lenalidomide						
				Dexamethasone						
										followed by TMP-SMX (15 mg/kg/day) and moxifloxacin (400 mg/day) 12 month
3	28	Female	SLE	Prednisolone	Headache Irritability nuchal rigidity	Cerebritis	Biopsy of Lesion	Right middle cerebral artery	Surgical excision of aneurysm with Bye pass	TMP-SMX 3 week
										Cefotaxime +amikacin
										2 weeks Doxycycline 4 weeks

**Table 2:** Reported cases of human infection with N otitidiscaviarum.

Authors/year of publication	Age/gender	Risk factors	Affected site	Imaging modality/findings	Treatment /duration	Steroid use	Outcome	Reference
Princess I et al. 2018	51/M	Asthma	Lung	consolidation of the left lung with destruction of the left lung	TMP-SMX + imipenem	+	Died	[20]
Tajima K et al. 2018	66/M	Lymphoma	Lung + Meninges	Multiple nodular lesions in the lung	TMP-SMX+ linezolid	–	Recovered	[21]
				The strain isolated from the CSF was identified as a *N. otitidiscaviarum*-type strain				
Thirouvengadame S Et al.,2017	65/M	None	Lung	infiltrative lesions in the middle zone of both lungs	TMP- SMX x 3 months	–	Recovered	[22]
Liu C et al. 2017	58/ M	hepatitis B virus Smoker	Lung	presence of nodules, masses, patchy consolidations, and bilateral pleural effusion	TMP-SMX + Amikacin + imipenem	–	Died	[23]
Sah R 2020	61/M	Nephrotic Syndrome	Lung + Skin	consolidation (mass-like lesion 3.5 × 3.5 cm) in the right upper lobe with right-sided pleural effusion and cystic lesion in the left upper lobe	TMP-SMX	+	Recovered	[24]
				USG of thigh: pus collection in the right thigh				
Sadamatsu H et al. 2017	72/F	Asthma	Lung	irregularly shaped solid opacity in the right middle lobe, a cavitary mass in the left lower lobe and bronchiectasis in both lower lobes	Minocycline 4 weeks	+	Recovered	[25]
					Levofloxacin 6 months			
Deepa R et al. 2016	14/F	Rheumatic heart disease	Lung	right lower lobe consolidation and right sided pleural effusion	Death Prior to identification	–	Died	[26]
Jiang Y et al. 2016	37/M	None	Lung + Liver + Neck mass	consolidation in the upper lobe of right lung, multiple nodules in both lung and right pleural effusion.	Minocycline TMP-SMX	–	Recovered	[9]
Simmons BP et al. 1992	60/M	Heart transplant	Lung + skin and soft tissue	Only abstract accessible	Imipenem/cilastatin and TMP-SMX and doxycycline	–	Recovered	[27]
Castelli et al*.*, 1994	31/M	HIV, Trauma	Skin and soft tissue + brain	Hypodensity in the right fronto- temporal and in the left temporo-parietal areas of probable inflammatory origin	TMP-SMX	–	Died	[28]
Clark et al*.*, 1995	86/M	Trauma	Skin	Vertebral Fracture	TMP-SMX x 10 weeks	+	Recovered	[29]
Suzuki et al*.*, 1995	78/F	Asthma	Skin + Lymph node	None	Minocycline + Doxycycline	+	Recovered	[30]
Mereghetti et al*.*, 1997	31/M	Trauma	Skin	There were no radiological sign of osteomyelitis.	Surgery, IV TMP-SMX +imipenem x 3 weeks. Then TMP-SMX x 3 weeks	–	Recovered	[31]
Sandre et al*.*, 1997	59/M	HIV	Chest wall + Lung + Abdomen	large septated extraperitoneal mass crossing both inside and outside the left thoracic and abdominal wall	Surgery, TMP/SMX + amikacin x 6 weeks	–	Recovered	[32]
Taniguchi et al*.*, 1998	76/M	Tuberculosis	Lung	Diffuse reticulonodular shadows on both lung fields	TMP-SMX x 6 months	–	Recovered	[33]
Hartmann et al*.*, 2000	50/F	Renal Transplant	Brain	a multilocular expanding process in the right frontal lobe with edema and some displacement of the midline structures	Meopenem + rifampicin X 6 weeks. Oral ciprofloxacin + rifampicin for 2 months.	+	Recovered	[34]
Duran et al*.*, 2001	21/M	IV drug	Brain	upper-posterior parietal right-sided cystic mass	imipenem + TMP-SMX	+	Recovered	[35]
Wada et al*.*, 2002	69/F	Trauma	Skin+ Lyph nodes	None	TMP-SMX	–	Recovered	[36]
					6 months			
Jennifer et al*.*, 2002	77/M	Rheumatoid arthritis, chronic bronchitis, Hypertension,trauma	Skin	None	Minocycline+ Clarithromycine 6 months	+	Recovered	[37]
Dikensoy et al*.*, 2004	65/M	None	Lung	multiple cavitary/non‐cavitary nodules throughout the right lung and a large consolidation consisting cavitating areas on the lower two‐third of the left lung	IV Amikacin + TMP-SMX x 1 month TMP-SMX 4 months	–	Recovered	[38]
Hemmersbach et al*.*, 2004	44/M	Renal Transplant, Diabetes	Brain	3 contrast enhancing lesions with enhancement localized in the frontal lobe, parietal lobe and the cerebellum	TMP-SMX 4 month	–	Died	[39]
Yoshida et al*.*, 2004	69/M	Rheumatoid arthritis	Lung	also showed the presence of fluid encap-sulated by an irregularly thickened pleural membrane	TMP-SMX + Levofloxacin +Gentamicin	+	Recovered	[40]
Fabre et al*.*, 2005	70/M	Rheumatoid arthritis, Infliximab	Skin	CT head and Lung Normal	Ofloxacin + Clindamycin	+	Recovered	[41]
Sharma et al*.*, 2007	36/F	Sickle Cell, End Stage Renal Disease	Lung Blood	multiple pulmonary nodules scattered throughout both lung fields	Amikacin + Gatifloxacin	–	Recovered	[16]
Thoms et al*.*, 2007	55/M	None	Skin	A computed tomographic (CT) scan of the left leg excluded any muscle or bone involvement	TMP-SMX + Amikacin Imipenem	–	Recovered	[42]
Pelaez et al*.*, 2009	85/F	Chronic Obstructive Pulmonary Disease	Disseminated. Lung brain	interstitial distribution of multiple pulmonary nodules, fluid in the major fissure, and a small pleural effusionThree nodular lesions in the left frontoparietal lobe with perilesional vasogenic edema,	TMP-SMX Imipenem Linezolid	–	Died	[43]
Betran et al*.*, 2010	57/M	Diabetes, Thrombocytopenia	Lung	consolidation throughout the lower right lobe and one nodule compatible with cavitary pneumonia.	TMP-SMX	–	Recovered	[44]
Chen et al*.*, 2011	51/M	None	Skin, Soft tissue	MRI revealed extensive inflammatory change with multiple focal fluid collections. Some of these lesions showed a central tiny hypointense focus, resulting in the dot‐in‐circle sign	TMP-SMX PO x 1year	–	Recovered	[45]
Ramamoorthi et al*.*, 2011	36/M	None	Lung	Peripheral pleural based thin walled cavitatory lesion with irregular inner margins, measuring 4.3 cm × 2.8 cm × 5.5 cm in the apico posterior segment of the left upper lobe.	TMP-SMX x 6 months	+	Recovered	[46]
Praveen et al*.*, 2014	60/M	Chronic obstructive pulmonary disease, trauma	Subcutaneous, soft tissue	Chest X-ray revealed increased bronchovascular markings in para-hilar region with emphysematous changes	Amikacin + linezolid for 4 weeks then Oral linezolid for 8 weeks	+	Recovered	[47]
Douedi S et al. 2020	56/M	Diabetes	Skin	venous thrombosis of cephalic vein and abscess formation within the soft tissue of right axilla	TMP-SMX for 3 months	–	Recovered	[48]
Scheelje Carabelli et al. 2019	89/F	Asthma, Bronchiectasis	Lung	right hydropneumothorax and areas of consolidation	cotrimoxazole, amikacin, + imipenem	+	Died	[49]
Paniagua-García M et al. 2019	57/M	Diabetes mellitus, Chronic obstructive pulmonary disease	Lung Brain Skin	Right pleural effusion with diffuse thickening of the pleural layers, suggestive of empyema	TMP-SMX Linezolid	–	Died	[50]
Saksena R et al. 2020	70/F	None	Lung	None described	TMP-SMX	–	Died	[51]
Magalhães GM et al. 2010	37/M	None	Hand	osteolytic lesion on the hand bones	TMP-SMX	–	Recovery	[52]
Yi-Chun Chen et al. 2013	61/M	Diabetes mellitus, Chronic liver disease	Lung	Pleural Effusion	No detail available	–	Died	[53]
Yi-Chun Chen et al. 2013	47/M	Diabetes Mellitus	Lung	Pleural Effusion	No detail available	–	Survived	[53]
Chung-Hao Huang et al. 2015	42/M	Cirrhosis	Lung	Pleural effusion	meropenem, TMP-SMX and amikacin.	+	Survived	[54]
Talwar P et al. 1989	50/F	None	Brain	Abscess	TMP-SMX +gentamicin, metronidazole	–	Died	[55]
Torre NP et al. 1991	75/M	Diabetes	Knee	Pleural Effusion	TMP-SMX + amoxycillin-clavulanate	–	Recovered	[56]
			Lung					
Mufti P et al. 1995	10 days /male	Home delivery	Lung	consolidation of left lung with pleural effusion and cavitation	cefotaxime and amikacin	–	Died	[57]
Eren E et al. 2016	69/F	None	Brain Abdomen	bilateral multiple hemispheric lesions	Meropenem+Amikacin +	–	Recovered	[58]
					TMP-SMX			
				located in right parietal lobe with an intense edema				
Alteras I et al. 1986	39/F	None	Foot	No involvement of the bone	Isoniazide Tetracycline	–	Recovered	[59]
Alteras I et al. 1980	41/M	None	Foot	No involvement of the bone	Isoniazide Tetracycline	–	Recovered	[60]
Min-Hui Chi 2012	71/M	SLE	Foot	None described	Tetracycline +Imipenam+ Meropenem +Amikacin	+	Died	[61]
Girouard Y et al. 1987	60/M	None	Finger	No osteomyelitis	TMP-SMX	–	Recovered	[62]
Hachisuka H et al. 1989	82/M	None	Hand	Chest Xray normal	oral chloramphenicol (1500 mg/day)		Recovered	[63]
Mahgoub A et al. 2016	41 /F	Asthma	Lung	bilateral asymmetrical patchy air space disease, consolidation on the middle lobe and bilateral pleural effusion, but no cavities	ceftriaxone 2 g IV twice daily +amikacin 500 mg IV twice daily + ciprofloxacin 500 mg orally twice daily.	–	Recovered	[64]
Salh B et al.	65/M	Diabetes Mellitus	Lung	Patchy consolidation	Gentamicin, Sulfadimidine	+	Recovered	[65]
		Liver Cirrhosis						
Yang LJ et al. 1993	63/M	Chronic Obstructive Pulmonary Diseases	Inguinal Lymphadenopathy	Enlarged lymph nodes in the left inguinal area,	oral TMP-SMX (80 and400mg), two tablets twice daily.	+	Recovered	[66]
Bradsher RW	69/M	Head Trauma	Brain	right-sided inferior frontal mass	sulfisoxazole	–	Died	[67]
Saarinen KA et al. 2001	50/M	None	Chest Wall Axilla	irregular skin thickening and massive enlargement of the soft tissue of the right side of the thoracic wall	rifampicin and TMP-SMX	+	Recovered	
				Pleural effusion and infiltration in the right lung were also observed				

There are many challenges in managing nocardial infection. On one hand, differentiating *Nocardia* species using biochemical characteristics is time-consuming and unreliable. On the other hand, genotypic methods of identifying N. spp including 16s ribosomal RNA sequencing and MALDI-TOF are fast, sensitive, and highly reliable. However, the non-availability of these facilities in many centers can delay the identification of causative agents. The treatment of *Nocardia* is challenging in the absence of prospective randomized trials to decide the most effective therapy for nocardiosis. The choice of antimicrobials is based on cumulative retrospective experience, results of investigations in animal models, and in vitro antimicrobial activity profiles. Antibiotics that are typically effective against N. spp include trimethoprim-sulfamethoxazole (TMP-SMX), amikacin, imipenem, and third generation cephalosporins (ceftriaxone and cefotaxime). However, antibiotic susceptibilities vary among isolates, and hence antimicrobial susceptibility testing is strongly recommended as there can be inter/intra-species variability in susceptibility patterns. Most of the frequently isolated species of *Nocardia* are found to be sensitive to TMP-SMX, imipenem, and linezolid and a combination of these agents may be used as empirical therapy. The Infectious Diseases Community of Practice of the American Society of Transplantation has published Guidelines on *Nocardia* infections in solid organ transplantation [69]. In the absence of other guidelines, the recommendations of this guideline can be extrapolated to other groups of patients with nocardiosis. They recommend TMP-SMX as first-line therapy in all patients with nocardiosis. Monotherapy is recommended for cutaneous as well as stable pulmonary disease. Imipenem, ceftriaxone, or linezolid are recommended agents when TMP-SMX cannot be used due to allergy or other causes. At least two agents (imipenem + amikacin or TMP‐SMX) are recommended for initial therapy in severe pulmonary infection, CNS involvement, and disseminated disease. This guideline states that the use of three drugs for life-threatening diseases can be considered as a weak recommendation. They recommend at least 12-month therapy for cerebral nocardiosis and to ensure resolution of lesions radiographically prior to stopping therapy. Surgical intervention may be needed in several settings in nocardiosis. For instance, cerebral abscesses, empyema, and mediastinal fluid collections are some conditions necessitating surgical intervention. Brain abscess of greater than 2.5 cm size is considered as an indication for aspiration. A clinical pathway published in 2014 recommends craniotomy for nocardial brain abscess in those with systemic infections and multiple brain lesions [70]. Literature shows few cases of *Nocardia* that had a benign course for years despite not receiving appropriate treatment [42], [45], [52], [68].

## Conclusion

Disseminated *N. otitidiscaviarum* is an uncommon presentation. This is the fourth reported case of intracranial aneurysm reported due to *Nocardia*. Possibility of intracranial aneurysm has to be considered while managing patients with nocardial infections.

Our patient presented relatively late, had a complicated course requiring multiple procedures, and a poor outcome despite proper antibiotics use and supportive care. Physicians managing patients with SLE need to be aware of the possibility of uncommon infections with unusual presentation. Patients with SLE should be educated about their immunosuppressed state and the need to present early to health care facilities.

## Funding

The authors received no financial support for the research, authorship, and/or publication of this article.

## CRediT authorship contribution statement

**Jabeed Parengal:** Conceptualization, Visualization, Writing – original draft, Data curation. **Seham Mohsin Alebbi:** data collection, data analysis, manuscript writing**. Manal Mahmoud Mohamed Hamed:** Investigation. **Hosam Mohammed Alqatami:** Investigation, Resources. **Fatma Ben Abid:** Conceptualization, Writing – review & editing, Supervision, Project administration.
